# Inflammatory Bowel Sugar Disease: A Pause From New Pharmacological Agents and an Embrace of Natural Therapy

**DOI:** 10.7759/cureus.42786

**Published:** 2023-08-01

**Authors:** Anas Mahmoud, Maha Begg, Mawada Tarhuni, Monique N. Fotso, Natalie A Gonzalez, Raghavendra R Sanivarapu, Usama Osman, Abishek Latha Kumar, Aishwarya Sadagopan, Michael Alfonso

**Affiliations:** 1 Internal Medicine, St. Joseph’s University Medical Center, Paterson, USA; 2 Internal Medicine, California Institute of Behavioral Neurosciences & Psychology, Fairfield, USA; 3 Pulmonary and Critical Care Medicine, Texas Tech University Health Sciences Center, Odessa, USA; 4 Pulmonary and Critical Care Medicine, Nassau University Medical Center, East Meadow, USA; 5 Geriatrics, Michigan State University College of Human Medicine, East Lansing, USA; 6 Internal Medicine, Spartan Health Sciences University, Vieux Fort, LCA

**Keywords:** metformin, breast milk, sun exposure, fecal microbiota implantation, vitamin, crohn’s disease (cd), probiotics and microbiome, time-restricted fasting, intestinal microbiota, inflammatory bowel diseases (ibd)

## Abstract

Inflammatory bowel diseases (IBDs), including Crohn's disease and ulcerative colitis, are immune-mediated chronic inflammatory diseases that target the gastrointestinal tract and other distant organs. The incidence of IBDs has been rising and is more prevailing in Western communities. The etiology has been vague, but different theories include environmental factors that elicit an uncontrolled immune response, which damages internal organs. Treatment of either Crohn's disease or ulcerative colitis has witnessed significant advances; however, pharmacological drugs' side effects limit their use. Research about microbiota and its influence on IBDs has gained fame, and multiple studies correlate microbiota diversity positively with IBD treatment. Many factors contribute to the microbiota's health, including different diets, antibiotics, prebiotics, probiotics, synbiotics, and postbiotics. Specific immune responses lie behind the pathogenesis of IBDs and microbiota dysbiosis, and different studies have postulated new ways to control this abnormal response. Physical activity, sun exposure, efficient sleep, intermittent fasting, and supplementation of probiotics and vitamins are natural ways that help modulate this immune response, do not cost money as IBD pharmacological drugs, and do not come with deleterious side effects that are sometimes more harmful than IBDs. Our article proposes a comprehensive natural approach that can benefit IBD patients enormously. This approach does not replace the medications currently used in treating IBDs. The suggested approach can be used in combination with medications and might aid in reducing the doses of those medications.

## Introduction and background

Ancient humans had a more robust gut and, consequently, less inflammatory-mediated metabolic diseases, which urged researchers to study microbiota extensively besides other natural ways that attributed the gut strength and tie those factors to different metabolic diseases, including inflammatory bowel diseases (IBDs). IBDs affect millions of people worldwide, but their exact cause yet remains unknown. One intriguing study area involves the role of the human microbiota, to which researchers hypothesize the rapid growth of IBD incidence. The refined sugar industry has grown over recent years and has made the incidence of inflammatory-mediated metabolic diseases grow higher as well; therefore, we entitled our article Inflammatory Bowel-Sugar Disease (IBSD) as it is proven that sugar plays a significant impact on gut inflammation. An unhealthy diet changes the microbiota's density and diversity, stripping the natural defense of the gut mucosa against inflammation. To better explain, the estimated number of microbiome genomes is believed to be a hundred times the amount of our genomes. This disruptive inflammatory reaction not only increases the incidence of IBDs but also increases the incidence of other diseases, such as diabetes mellitus (DM). The microbiota is also famous for secreting short-chain fatty acids (SCFAs), which have enormous benefits in regulating gut inflammation. Fecal microbiota transplantation (FMT) has effectively treated intestinal infections and other non-gastrointestinal diseases such as autism and seizures. These profound benefits of the microbiota have also been evident in healthy infants, as breast milk significantly affects the microbiota by increasing its diversity and propensity, resulting in less inflammation and better overall immunity. Many physicians are skeptical about natural medicine, e.g., probiotics, and lean back to traditional pharmacological drugs and rather wait for natural medicine supplements to be studied extensively and listed as guidelines. Probiotics notably affect gut inflammation through inhibition of tumor necrosis factor-α (TNF-α)-related cytokines and stimulation of interleukin-10. Supernatants, a form of postbiotics, increase the intestinal peristalsis caused by stress. Vitamins can play a role in IBD inflammation; for instance, vitamin D increases microbiota diversity and modulates gut inflammation by regulating several inflammatory pathways. Following our ancestors' lifestyles, researchers have proven the benefits of prolonged fasting hours and limited food intake on gut microbiota and the substantial significance on body inflammation. Sun exposure, night sleep, stress-free lifestyle, and exercise play consequential roles in immune modulation and inflammatory marker regulation. This article explains the inflammation process behind developing IBDs and tries to put many suggested treatments into a comprehensive natural approach, as an addition to currently used pharmacological medications, to prevent, treat, and reverse inflammatory gut in IBDs. 

## Review

IBDs, the umbrella under which two major disorders fall: Crohn's disease (CD) and ulcerative colitis (UC), present as chronic relapsing disorders of the gastrointestinal (GI) tract. The etiology of IBDs is not known yet; however, recent data have shed light on some environmental factors that can trigger specific genes, which could lead to uncontrolled mucosal immune reactions and significant damage to the GI and other extra-intestinal organs [1]. The imbalance of any of three different factors can increase the risk of developing IBD: genes, the host immune system, and environmental factors. Firstly, ninety-nine susceptibility genes are related to IBDs via genome-wide association studies. Interestingly, among those genes, many are also associated with the risk of inflammatory-mediated metabolic diseases, including type 1 and 2 DM [2]. Secondly, environmental factors such as the microbiota are vital for gut health, and any disturbance to the microbiome increases the risk of many gut pathologies, including IBDs. For instance, Armstrong et al. found that mutated mice lacking IL-10 had dysbiosis, a microbiome imbalance, and consequently an increased risk of developing IBDs [3]. Thirdly, an abnormal immune response against the intestinal mucosa can elicit an inflammatory cascade that destroys the gut mucosa. Hence, understanding and modulating the immune response can prevent and treat IBDs. Since IBDs are immune-mediated inflammatory diseases, this article will discuss different natural methods, including fasting, sun exposure, physical activity, healthy food, supplementations with vitamins and probiotics that can modulate this immune reaction, and the approach of a comprehensive natural model that could tackle IBDs. The authors will present each suggested natural approach separately and its correlation with IBDs in evidence-based medicine facts. 

Microbiota and IBDs

First, we will start with the most recent data about microbiota and how it could affect IBDs. Gut microbiota refers to the bacteria, eukarya, and archaea that colonize the GI tract, in which a delicate equilibrium between those microorganisms ensures gut health. Enriched microbiota diversity improves lipid profiles and intense anti-inflammatory modulations, leading to better metabolic functions and overall health [4]. The average number of gut microbes is estimated to be over 100 times the amount of genomic content (microbiome) than the human genome. Isolation of microbiota from the small intestine and colon showed different organisms, where *Lactobacillaceae* is the main dominant species in the small intestine, and *Prevotellaceae*, *Lachnospiraceae*, and *Rikenellaceae* dominate in the colon in experimental mice [5]. Any disruption to this balance is called dysbiosis, which could increase the risk of developing diseases such as IBDs and DM.

Microflora commensals are short-lived and are easily affected by chemicals, infections, or chronic inflammation, which could lead to disequilibrium of the microbiota in number or diversity, and either defect is called dysbiosis. For example, poor microbiota diversity, such as *Firmicutes*, provides less energy for the intestinal epithelium to grow and differentiate. Fewer numbers of SCFA-producing bacteria, e.g., a decrease of clostridium cluster IV, XIVA, XVII, and *Faecalilbacterium* *prausnitzzi* could result in dysregulation of T-cell differentiation, thus a higher risk for IBDs, autoimmune hepatitis [6], and IgG4-sclerosing cholangitis [7]. Not only diversity and numbers but any increased activity of a mucolytic bacteria such as *Ruminococcus* *canvas* and *Rumniococcus* *torques* could increase the bacterial invasion due to poor mucous layer. Microbiota has different species which can be harmful or benign, and in some instances, harmful bacteria could overgrow the benign ones and cause damage; for example, sulfate-reducing bacteria such as *Desulfovibrio* could induce gut wall inflammation and have been isolated in colonic microbiota in IBD patients.

To better associate microbiota with IBDs, researchers have studied how the IBD affects the microbiome and creates dysbiosis, translating into gut damage. In IBDs, a higher portion of *Proteobacteria, Pasteurellaceae, Ruminococcus gnavus, Veillonellaceae, Fusobacteria, Candida tropicalis,* and decreased amount of *Firmicutes* (especially the potentially protective *Faecalibacterium* *prausnitzii*), *Bifidobacterium*, *Ruminococci*, and *Clostridium* have shown [8]. 

Microbiota are not only vital for the gut system but have benefits in distant organs. For instance, *Lactobacillus Plantarum* P8 ameliorates memory and cognition; *Clostridium butyricum* restores hippocampal microglial activation; *Prevotella*, *Prevotellaceae*, *Bacteroidia*, and *Dialisster* adjust the circadian system and improve intestinal physiology and metabolism;*Lactobacillus rhamnosus (JB-1)* helps with stress and anxiety; *Oscillibacter spp.* and *Lactobacillus spp.* ameliorate the metabolism of glucose and lipids; *Lactobacillus* and *Faecalibacterium prausnitzii* modulate inflammation; and *Bifidobacterium* has weight-loss benefits [9]. 

**Figure 1:**
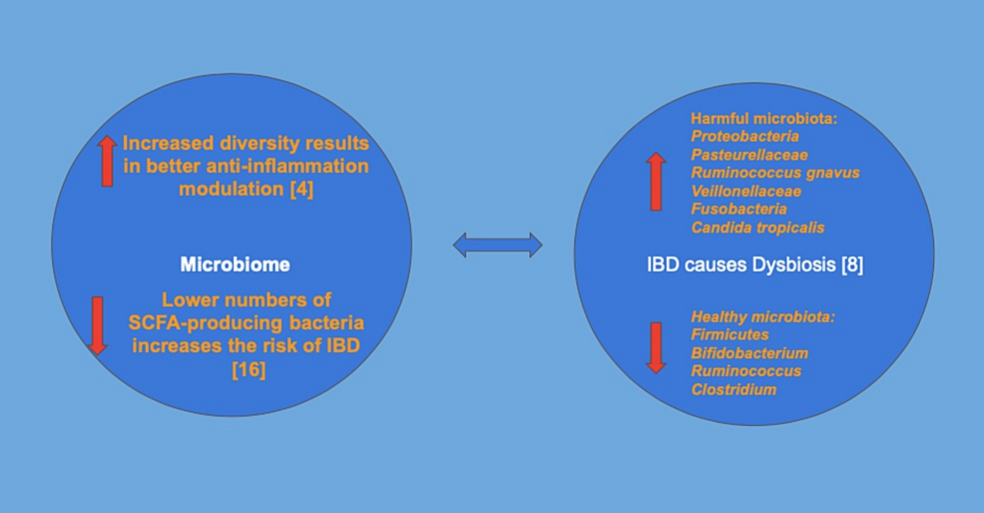
IBD and microbiome interaction The left figure explains how microbiota affects the IBD; better diversity of microbiota results in better modulation of the anti-inflammatory reactions in the gut which subsequently attenuates IBD pathogenesis [4]. Also weak microbiota yields in lower numbers of SCFA-producing bacteria which results in a higher IBD risk. The right figure explains how the IBD itself causes dysbiosis and weakens the microbiota. The IBD increases the number of harmful microbiota species such as *Proteobacteria, Pasteurellaceae, Ruminococcus gnavus, Veillonellaceae, Fusobacteria, and Candida tropicalis* and decreases the number of healthy beneficial microbiota such as *Firmicutes, Bifidobacterium, Ruminococci, and Clostridium* [8] This figure is the author’s own creation. IBD: Inflammatory bowel disease; SCFA: short-chain fatty acid

Due to the above-listed benefits of microbiota, many researchers and clinicians studied the efficacy of implantation of microbiota in different gut diseases; for example, Zeng et al. launched a meta-analysis regarding fecal microbiota transplantation (FMT) and established its efficacy and safety in treating IBDs [10]. He et al. also performed a pilot randomized controlled study on FMT on maintaining remission in IBDs, which was statistically significant [11]. FMT has also successfully improved mentation in cirrhotic patients, behavior in autistic individuals, and seizures. Figure 1 summarizes the interconnection between the microbiome and IBD.

Breast milk, microbiota, and IBDs

Breast milk is believed to play a significant role in the IBD as human milk oligosaccharides (HMOs) have a similar structure to the intestinal mucin and can outcome the bacterial mucus-degrading CAZymes which play a role in mucous catabolism and can prevent inflammation [12]. *Bifidobacterium longum* and *Bacteroides* are more abundant in breast milk and can enrich microbiota composition more than *Escherichia coli *and *Clostridium* *perfringens* in formula milk. The microbiota isolated in African infants produced more SCFAs, which have a significant role in inflammation in the gut. On the contrary, European infants and adults who consume less microbiota-accessible carbohydrates, aka MAC diet (rich in complex carbohydrates), have less SCFA content and more risk of IBDs. Therefore, the healthier breastfed infants, compared to their formula-fed counterparts, have stronger microbiota, fewer infections, lower risk for IBDs, and better immunity.

Different diets can influence the gut microbiota, where the ileal microbiota could be affected by sugar. Meanwhile, dietary fibers target the colonic microbiota. Walker et al. studied the influence of dietary fibers on the integrity of the gut system through enhancing the microbiota, and according to them, diets with abundant resistant starch or in non-starch polysaccharide fiber yielded more robust and reproducible enrichment of different bacterial species in the human gut compared to other diets [13]. *Bacteroides*, for instance, are more abundant in the rural African diet, which has more fiber, compared to the European diet, which has more sugar.

Administration of the MAC diet successfully restored the healthy gut microbiota in mice and attenuated gut inflammation. Gut microbiota commences on carbohydrates provided by the intestinal mucus with inner and outer layers and is thickest in the colon epithelium. A low MAC diet resulted in thinner mucus in the distal colon which increased the proximity of microbes to the epithelium and heightened expression of the inflammatory marker REGIIIβ, contributing to more inflammation [14]. 

Prebiotics, probiotics, microbiota, and IBDs

Prebiotics, probiotics, and synbiotics (i.e. a mixture of pre and probiotics) have been suggested for treating IBDs by strengthening the intestinal mucosa by restoring healthy microbiota and modulating their interaction and the host immune defense [15]. Prebiotics (lactulose, lactosucrose, HMO, inulin, and others) promote the growth of specific microbiota, which modulates the inflammatory status in IBDs. Probiotics (most commonly used: *Lactobacillus *species, *Bifidobacterium* species, *Streptococcus*, as well as yeast *Saccharomyces*) can release specific antimicrobial mediators (lactic and hydrogen peroxide, acetic acid, and bacteriocins) which protect against different gut pathogens. Probiotics modulate inflammation by inhibiting the expression of tumor necrosis factor-α (TNF-α)-related cytokines while augmenting interleukin-10 levels. It is very critical to take the approved dosage of probiotics as well as the purity and condensation of probiotics supplementation. 

Jiang et al. have found that supplementation with *Lactobacillus rhamnosus M3*, a lactic acid bacterium, was associated with enhanced gut microbiota's antibacterial activity against* Staphylococcus aureus* and *Pseudomonas aeruginosa*, *Escherichia coli,* and *Shigella *[16]. The enhanced antibacterial activity of *Lactobacillus rhamnosus M3* was achievable through its better adherence to the gut mucosa, pH reduction, and SCFA production, as well as increased acetic acid and propionic acid levels. They also suggested supplementing milk with *L *Lactobacillus *rhamnosus M3* to restore powerful gut microbiota. *Ruminococcus gnavus* can provide trans-sialidase to the gut commensal bacteria, which can help against many pathogens such as *Salmonella typhimurium *and *Clostridium difficile*, which lack a sialidase, which resulted in less gut inflammation. *Muciniphila* supplementation has gained popularity in treating obesity and IBDs by enhancing the microbiota health as it restores the strong gut barrier, which helps prevent the development of high-fat diet-induced obesity and ameliorates metabolic endotoxemia-induced inflammation. *Lactobacillus fermentum ZS40* supplementation in mice has shown an effect in preventing colon cancer through decreasing levels of CD34, CD117, TNF-α and IL-1β, IκBα, and p65 in the NF-κB signaling pathway, which can lead to a reduction of other malignancies as higher levels of CD117 are linked to acute promyelocytic leukemia, for instance [17]. 

Postbiotics, microbiome, and IBDs

Żółkiewicz et al. have studied postbiotics and defined them as any substances of bacterial or fungal origin that have beneficial effects and do not meet the definition of a probiotic or prebiotics [18]. Here, we list five subgroups of probiotics that have applications in IBDs.

Cell-free supernatants produced from *Lactobacillus acidophilus* and *Lactobacillus* *casei* can reduce the secretion of (TNF-α) and increase the secretion of IL-10. In contrast, the ones derived from *Lactobacillus casei* and *Lactobacillus rhamnosus* can prevent the invasion of colon cancer cells, and the ones derived from *Lactobacillus* and *Bifidobacterium* can prevent the invasion of enteroinvasive *Escherichia coli*, which can help in diarrhea, and *Lactobacillus plantarum* has a positive effect on intestinal barrier and decreases TNF-α and IL-1β. Supernatants from *Saccharomyces cerevisiae* and *Saccharomyces boulardii *could reverse intestinal peristalsis caused by stress [19]. Microorganisms produce biopolymers released outside bacterial cell walls forming exopolysaccharides (EPSs). EPSs modulate the immune response through the regulation of T and NK lymphocytes. EPSs from *Lactiplantibacillus plantarum* and tofu induced nitric oxide (NO) secretion, while EPSs from *Lactobacillus helveticus*, as in green tea, can bind iron ions and exert antioxidant activity. EPSs produced by *Lactobacillus kefiranofaciens* from kefiran consumption delayed the development of atherosclerosis in rabbits [20]. β-glucans, another class of EPSs, could facilitate the adhesion of lactobacilli to the intestinal epithelium and increase the absorption of carotenoids offering antioxidant and anti-inflammatory properties to the gut mucosa. Enzymes, as a form of postbiotics, can attenuate inflammation in the gut system. *Lactobacillus fermentum* and *Lactiplantibacillus plantarum* produce high amounts of glutathione peroxidase and peroxide dismutase, evident in decreasing inflammation in CD in mice [21]. *Lactobacillus lactis *offered higher catalase activity, preventing chemically induced colon cancer in mice. Microbiota can produce SCFAs by fermentation of polysaccharides. SCFAs include acetic, propionic, and butyric acids, which can form the corresponding fatty acid salts (i.e., acetate, propionate, and butyrate) [22]. *Roseburia intestinalis* produce butyrate which helps renew intestinal epithelium by inhibiting histone deacetylase, increasing type 1 interferon, IL-10, TGF-β, and down-regulating several cytokines and pro-inflammatory receptors (e.g., toll-like receptor (TLR), caspase-1, NLRP3, IL-1β, IL-18, IL-33, IL-25, MAPK, and NF-κB1 activity, with ultimate reduction in prominent intestine inflammation in IBDs. Acetate decreased insulin resistance by increasing GLP-1 levels and increased resistance to enterohaemorrhagic* Escherichia coli* in mice by sealing the intestinal barrier against lethal toxin entry. Propionate also exerts some anti-inflammatory activity against demyelination and was able to provide symptom relief in mice with multiple sclerosis. Finally, metabolites of microbiota offer significant health benefits. Foliate produced by *Lactobacillus helveticus CD6 *can ameliorate DNA synthesis and reparation and offer antioxidants. Supplementation of *Lactobacillus acidophilus* in yogurt was associated with higher vitamin b12 levels which are low in CD [23]. Microbiota can metabolize polyphenols and secrete urolithin A, equol, and 8-prenylnaringenin (8-PN), ameliorating sugar and fatty acid metabolism and enhancing insulin sensitivity. Table 1 summarizes the supplementation of prebiotics, probiotics, and postbiotics.

**Table 1:** Prebiotic, probiotic, and postbiotic supplementation and their effects on microbiome and IBD This table summarizes the above-mentioned effects of supplementation of prebiotics, probiotics, and postbiotics. Postbiotics are listed in five different categories. IBD: Inflammatory bowel disease

Supplement	Effect on microbiome and IBD
Prebiotics	- Includes (lactulose, lactosucrose, HMO, inulin, and others). - Promotes diversity and strength of gut microbiota and helps attenuate inflammation in IBD [15].
Probiotics	- Most commonly used: Lactobacillus species, Bifidobacterium species, Streptococcus, Saccharomyces). - Release antimicrobial mediators (lactic and hydrogen peroxide, acetic acid, and bacteriocins) which protect against different gut pathogens (Staphylococcus aureus, Pseudomonas aeruginosa, and others) [16]. - Inhibit the expression of (TNF-α)-related cytokines, and augment IL-10 levels.
Postbiotics	
1- Cell-free supernatants	Depends on the source of microbiome: From Lactobacillus acidophilus and Lactobacillus casei can reduce the secretion of (TNF-α) and increase the secretion of IL-10 [18]. - From Lactobacillus casei and Lactobacillus rhamnosus can prevent the invasion of colon cancer cells. - From Lactobacillus and Bifidobacterium can prevent the invasion of enteroinvasive Escherichia coli, which can help with diarrhea. - From Lactobacillus plantarum has a positive effect on the intestinal barrier and decreases TNF-α and IL-1β.
2- Supernatants	Produced from Saccharomyces cerevisiae and Saccharomyces boulardii could reverse intestinal peristalsis caused by stress [19].
3- Exopolysaccharides (EPSs)	- From Lactiplantibacillus plantarum and tofu induced nitric oxide (NO) secretion, - - From Lactobacillus helveticus, as in green tea, can bind iron ions and exert antioxidant activity. - From Lactobacillus kefiranofaciens from kefiran consumption delayed the development of atherosclerosis in rabbits [20]. - β-glucans could facilitate the adhesion of lactobacilli to the intestinal epithelium and increase the absorption of carotenoids offering antioxidant and anti-inflammatory properties to the gut mucosa.
4- Enzymes (form of postbiotics)	- Lactobacillus fermentum and Lactiplantibacillus plantarum produce high amounts of glutathione peroxidase and peroxide dismutase, evident in decreasing inflammation in CD in mice [21] - Lactobacillus lactis offered higher catalase activity, preventing chemically induced colon cancer in mice. - SCFAs include acetic, propionic, and butyric acids, which can form the corresponding fatty acid salts (i.e., acetate, propionate, and butyrate) [22]. - Roseburia intestinalis produce butyrate which helps renew intestinal epithelium by inhibiting histone deacetylase, increasing type 1 interferon, IL-10, TGF-β, and down-regulating several cytokines and pro-inflammatory receptors (e.g., toll-like receptor (TLR), caspase-1, NLRP3, IL-1β, IL-18, IL-33, IL-25, MAPK, NF-κB1 activity, with ultimate reduction in prominent intestine inflammation in IBD. - Acetate decreased insulin resistance by increasing GLP-1 levels and increased resistance to enterohaemorrhagic Escherichia coli in mice by sealing the intestinal barrier against lethal toxin entry. Propionate also exerts some anti-inflammatory activity against demyelination and was able to provide symptom relief in mice with multiple sclerosis.
5- Metabolites of microbiota	- Foliates produced by Lactobacillus helveticus CD6 can ameliorate DNA synthesis and repair and offer antioxidants. - Supplementation of Lactobacillus acidophilus in yogurt was associated with higher vitamin B12 levels which are low in Crohn's disease [23] - Microbiota can metabolize polyphenols and secrete urolithin A, equol, and 8-prenylnaringenin (8-PN), ameliorating sugar and fatty acid metabolism and enhancing insulin sensitivity.

Vitamins, microbiota, and IBDs

Vitamins can play a role in IBDs through different mechanisms, including immunologic modulation and microbiota. Vitamin D deficiency reduces microbiota diversity and exacerbates inflammation in IBDs and was associated with a worse prognosis [24]. Vitamin D can increase IL2 levels, modulate T cell cytokine production, and improve the function of Foxp3+ Treg cells. Vitamin D receptor inhibits NF-κB activation and controls the IBD risk gene ATG16L1 and, therefore, can attenuate colon inflammation by regulating the JAK/STAT pathway. Vitamin A is suggested for use in treating IBDs through its active metabolite retinoic acid, which could inhibit IL-6, IL-17, INF-γ, and TNF-α, interact with TGF-β to increase the level of Foxp3, and protect the intestinal mucosal barrier by minimizing the destruction done by LPS. Vitamin B12 deficiency contributes to a higher degree of inflammation in mice [25] due to the over-expression of pro-inflammatory cytokines like TNF-α. 

Vitamins also interact with microbiota and influence the progression of IBDs. While four microbiota (*Firmicutes, Proteobacteria, Bacteroides, Actinomycetes*) can synthesize vitamins (mainly riboflavin and niacin) in the gut, supplementation with oral vitamins (B2, C, D) has been shown to increase microbiota diversity. Vitamin A supplementation has proven to help in IBDs as it increased *Akkermansia, Lactobacillus, Prevotella, Aerococcus* and decreased *Bacteroides, Parabacteroides, Escherichia/Shigella, Klebsiella, Oscillibacter, Pseudolavonifractor, Clostridium sensu stricto, Butyrimimonas, Mucispirllum, *and *Clostridium XIVb*. Vitamin B supplements increased *Actinobacteria and Odoribacteraceae* and decreased *Campylobacteraceae, Fusobacteriaceae, *and *Prevotellaceae*. Vitamin C increased *Lactobacillus sp*. and decreased *Enterobacteriaceae*. Vitamin D enhanced *Actinobacteria* and *Prevotella* growth and suppressed *Bacteroidetes, Veillonella, and Haemophilus* [26]. Vitamin E enhanced *Bacteroides and* *Proteobacteria* while vitamin E deficiency was associated with lower levels of *Ruminococcus, Lachnospiraceae, *and* Muribaculaceae*. It is very important to take vitamin supplementations under the supervision of physicians as over intake of vitamins is associated with toxicity of some vitamins. Table 2 summarizes the effect of breast milk and vitamins on microbiota and IBDs. 

**Table 2:** Summary of the effect of breast milk and different vitamins on microbiota and IBD This table summarizes the effect of breast milk and vitamins on microbiota diversity and strength and the effect on the IBD inflammatory process and markers IBD: Inflammatory bowel disease; SCFA: short-chain fatty acid; HMOs: human milk oligosaccharides

Breast milk and different vitamins	Effect on microbiome and IBD
Breast milk [12]	- HMOs (similar to intestinal mucin) overcome mucous-degrading CAZymes and prevent gut inflammation. - Increases beneficial microbiota: Bifidobacterium longum and Bacteroides. - Produce more SCFA content that modulates gut inflammation.
Vitamins [24,25,26]	Four microbiota (Firmicutes, Proteobacteria, Bacteroides, Actinomycetes) can synthesize vitamins (mainly riboflavin and niacin) in the gut. - Supplementation with oral vitamins (B2, C, D) has been shown to increase microbiota diversity
Vitamin D	- Vitamin D deficiency reduces microbiota diversity and exacerbates inflammation in IBD and was associated with a worse prognosis. - Increases IL2 levels, modulates T cell cytokine production, improves the function of Foxp3+ Treg cells, inhibits NF-κB activation, and controls the IBD risk gene ATG16L1 and, therefore, can attenuate colon inflammation by regulating JAK/STAT pathway.
Vitamin A	- Vitamin A active metabolite retinoic acid could inhibit IL-6, IL-17, INF-γ, and TNF-α, interact with TGF-β to increase the level of Foxp3, and protect the intestinal mucosal barrier by minimizing the destruction done by LPS. - Supplementation can increase healthy microbiota (Akkermansia, Lactobacillus, Prevotella, Aerococcus) and decrease pathogenic microbiota Bacteroides, Parabacteroides, etc.)
Vitamin B12	- Vitamin B12 deficiency contributes to a higher degree of inflammation in mice due to the over-expression of pro-inflammatory cytokines like TNF-α. - Vitamin B supplements increased Actinobacteria, Odoribacteraceae and decreased Campylobacteraceae, Fusobacteriaceae, and Prevotellaceae.
Vitamin C	- Increases Lactobacillus sp. and decreases Enterobacteriaceae
Vitamin E	- Increases Bacteroides and Proteobacteria. - Vitamin E deficiency was associated with lower levels of Ruminococcus, Lachnospiraceae, and Muribaculaceae.

Fasting, microbiota, and IBDs

Prolonged hours of fasting have a direct proportional relationship with gut microbiome numbers and richness. Our ancestors used to fast for days and happened to have less incidence of IBDs and other immune-mediated pathologies. Prolonged fasting increased the abundance of *Bacteroidetes and Prevotellaceae, Lachnospiraceae, Parasutterella, and Romboutsia* and the production of SCFAs. It was also found that the number of fasting hours resulted in different ratios of microbiota *Prevotella, Faecalibacterium, Bacteroidetes, and Firmicutes, Akkermansia muciniphila, Faecalibacterium prausnitzii, Bifidobacterium spp., Lactobacillus spp., Bacteroides fragilis group, and Enterobacteriaceae*. For instance, *Akkermansia muciniphila* supplementation as probiotics has been found to be effective as a therapeutic target in the microbiota‐related diseases, such as colitis, metabolic syndrome, immune diseases and cancer.

Time-restricted feeding (TRF) has been proven to control IBD activity's inflammatory response and attenuation. TRF enhances circadian clock oscillations, which results in better regulation of some metabolic regulators such as CREB (cAMP response element-binding protein), AMPK, mTOR, PPARg (peroxisome proliferator-activated receptor gamma), and PGC-1 alpha (peroxisome proliferator-activated receptor gamma coactivator 1-alpha) that control the levels of SIRT1, which could help against DNA damage and promote longevity [27]. Prolonged fasting is vital in controlling inflammation and decreasing TNF-a, IL-1, IL-6, and pro-inflammatory cytokines. TRF can result in better hormonal signaling, a significant increase in adiponectin, and an evident decrease in leptin and T3. TRF reduces adipose tissue burden, which secretes the most critical pro-inflammatory cytokines (TNF-α and IL-1). 

Sun, microbiota, and IBDs

Exposure to UVB light affects gut microbiota as it increases the numbers of *Firmicutes* (*Lachnospiraceae, Ruminococcus, and Clostridiaeae*), *Proteobacteria*, and *Verrucomicrobia* and decreases *Bacteroidetes*. Sun exposure increases vitamin D, which has a beneficial effect on gut microbiota. Nerich et al. have found that low sun exposure was associated with a higher incidence of CD [28].

Exercise, microbiota, and IBDs

Physical activity has decreased incidence, improved symptoms, delayed progression, and decreased mortality in IBDs. Exercise has been shown to modulate pro-inflammatory cytokine release and IL-6 and increase NK and T lymphocytes [29]. IL-6 can exert pro and anti-inflammatory effects depending on the body's environment, and interestingly, exercise drives IL-6 in the anti-inflammatory direction through increasing IL-10, cytokines, and irisin and decreasing TNF-α and leptin, while inactive status increases TNF-α. IL-6 up-regulation, in some COVID-19 patients has led to higher complications of cardiovascular disease and autonomic dysfunction [30]. Some studies have compared the microbiota and IBD activity with different durations and types of exercises; however, more studies are needed to determine the minimum duration and type of physical activity needed to produce effects on microbiota and IBD activity.

Exercise also plays a role in microbiota diversity and richness. Microbiota isolated in high physical activity adults showed higher SCFA producers like (*Clostridiales, Eubacterium, Blautia, Ruminococcaceae, and Faecalibacterium*), bile acids, and tryptophan. Hockey players have high levels of *Bifidobacterium animalis*, *Lactobacillus acidophilus, Prevotella intermedia, *and* F. prausnitzii*. Exercise can also decrease pro-inflammatory microbiota, such as *Bilophila* and *Faecalicoccus*. Thus, the increase in anti-inflammatory and decrease in pro-inflammatory microbiota associated with exercise can regulate inflammation in IBD patients [31].

Sleep, microbiota, and IBDs

Sleep efficiency possesses a positive correlation with microbiome diversity and IL-6 (a putative somatogenic factor and sleep regulator), and sleep fragmentation has a negative correlation as well; therefore, FMT may be a solution to improve sleep efficiency through BAGMA (brain-gut-microbiome axis). *Bacteroidetes* and *Firmicutes* positively correlated with sleep efficiency, while bacteroids negatively correlated with sleep fragmentation. *Bacteroidetes*, *Actinobacteria*, and *Firmicutes* produce γ-aminobutyric acid (GABA), a neurotransmitter that promotes sleep. SCFA producers *Lachnospiraceae* family, including *Blautia*, *Coprococcus*, and *Oribacterium,* had a negative correlation with sleep quality. Poroyko et al. reported poor numbers and quality of *Actinobacteria* in gut microbiota in mice with poor sleep quality [32]. Selection of diet components can affect the quality of sleep, for instance, diets rich in carbohydrates with high glycemic index increase the risk for insomnia. 

On the other hand, sleep impairment represents profoundly in IBD patients and yields more severe complications in IBD patients. Sleep disturbance with circadian rhythm imbalance might induce flares in IBDs. Table 3 summarizes the effects of previously mentioned natural therapies (fasting, sun exposure, exercise and sleep) on microbiota and IBDs.

**Table 3:** Effects of fasting, sun exposure, exercise, and sleep on microbiota and IBD This table points out the important effects of different natural techniques including fasting, sun exposure, exercise, and sleep on microbiota and IBD IBD: Inflammatory bowel disease; SCFA: short-chain fatty acid; CD: Crohn's disease

Natural approach	Effect on microbiota and IBD
Fasting [27]	- Increased the abundance of Bacteroidetes and Prevotellaceae, Lachnospiraceae, Parasutterella, and Romboutsia and the production of SCFAs. - Better regulation of inflammatory mediators (CREB, AMPK, mTOR, PPARg and PGC-1 alpha) that control the levels of SIRT1, which could help against DNA damage and promote longevity. - Decreases TNF-a, IL-1, IL-6, and pro-inflammatory cytokines. - Better hormonal signaling, a significant increase in adiponectin, and an evident decrease in leptin and T3.
Sun exposure [28]	- Increases numbers of Firmicutes (Lachnospiraceae, Ruminococcus, and Clostridiaeae), Proteobacteria, and Verrucomicrobia and decreases Bacteroidetes. - Increases vitamin D, which has a beneficial effect on gut microbiota. - Low sun exposure is associated with a higher incidence of CD.
Exercise [29,30,31]	- Modulates pro-inflammatory cytokine release and IL-6 and increases NK and T lymphocytes. - IL-6 induced by exercise can increase IL-10, cytokines, and irisin and decrease TNF-α and leptin. - Results in higher SCFA producers like (Clostridiales, Eubacterium, Blautia, Ruminococcaceae, and Faecalibacterium), bile acids, and tryptophan. - Decreases pro-inflammatory microbiota, such as Bilophila and Faecalicoccus.
Sleep [32]	- Associated with better microbiome diversity and higher IL-6 levels (a putative somatogenic factor and sleep regulator). - Bacteroidetes, Actinobacteria, and Firmicutes produce γ-aminobutyric acid (GABA), a neurotransmitter that promotes sleep.

Oral anti-diabetic medications, microbiota, and IBDs

DM and IBDs have grown in recent decades and are more evident in Western communities. Identifying the correlation between them can help understand the mechanism of developing and treating IBDs, given that the gut system is the central regulatory system in glucose absorption and utilization, and any disruption can lead to the development of either disease. Researchers have investigated IBD-related genes, and among those genes, many are also associated with the risk of metabolic diseases, including type 1 and 2 DM [2].

Metformin is the most commonly prescribed medication for patients with type 2 DM. Metformin's effect on the immune system in different organs has been studied extensively after its success in reducing resistance against insulin in diabetic patients. Recent studies in cell cultures and animals have suggested that metformin may reduce pro-inflammatory cytokines and chemokines in the intestine, protecting against intestinal barrier dysfunction [33]. Metformin also has anti-inflammatory signals as it suppresses lipopolysaccharide (LPS)-induced inflammatory response in cultured macrophages and endothelial cells and down-regulates inflammation in many organs [34]. Metformin suppresses ATP production by inhibiting mitochondrial complex one and glycerophosphate dehydrogenase, which can ultimately increase the AMP/ATP ratio and subsequently enhance the AMP-activated protein kinase (AMPK) activation. In genetically mutated mice where the AMPK gene was deleted, metformin administration yielded no actions on immune response in different organs, including insulin resistance [35].

On the contrary, metformin given to non-mutated mice statistically decreased colon inflammation due to increased expression of active AMP-activated protein kinase (AMPK) [36]. AMPK helps make a natural protective mucosal layer in the intestine against toxins which leads to a healthy gut; therefore, loss of AMPK destroys this protective mechanism and increases the incidence of diarrhea [37]. AMPK can down-regulate cytokines and other pro-inflammatory mediators in different auto-immune diseases such as auto-immune encephalomyelitis [38], antigen-induced arthritis, LPS-induced acute lung and heart injury [39]. Aside from the AMP-APK pathway, metformin suppresses p38 map kinase activation and inhibits IL-6 expression, which contributes to pathogenesis in gut tissue damage. Metformin ameliorates mouse colonic inflammation and protects gut epithelium in IL-10-deficient mice [40]. Metformin has been theoretically suggested to be used in patients with IBDs [41]. Chin-Hsiao Tseng studied over three hundred thousand diabetic patients and concluded that metformin is associated with a reduced risk of IBDs in a dose-response pattern. Surprisingly, this effect potentiates in patients using other oral anti-diabetics or insulin [42]. 

Metformin has shown influence on the microbiome by increasing the production of SCFAs and tryptophan, modulating the action of vitamin D, and increasing the production of ketone bodies and glucagon-like peptides (GLP) in the gut. Metformin decreased *Bacteroides fragilis* and increased mice's bile acid glycoursodeoxycholic acid (GUDCA) [43]. GUDCA antagonizes intestinal farnesoid X receptor FXR, which improves glucose metabolism in the gut [44]. Metformin also increased bile flow (bile acids and bilirubin) in the ileum of mice, and those bile acids can inhibit FXR-fibroblast growth factor; however, they also cause diarrhea in patients treated with metformin. Metformin could also cause undesirable effects on the gut microbiome, which accounts for the unpleasant gastrointestinal side effects of metformin. Metformin increases the genus *Escherichia* which subsequently increases gas metabolism and causes bloating. Hence, a gastrointestinal microbiome modulator was administered in conjugation with metformin in metformin-intolerant diabetic patients, improving metformin tolerance even with a higher metformin dose [45]. 

Adding SGLT-2 inhibitors, empagliflozin, to metformin has been shown to improve colitis through better regulation of inflammatory response. The administration of both agents together significantly augmented AMPK and depressed mTOR and NLRP3 expression, which resulted in a subsequent activity of caspase-1 cleavage and inhibition of different cytokines, including IL-1β, and IL-18, IL-6 with a reduction in Th17 activity. The effect of concomitant administration of medications was evident in lower disease activity and subsequent tissue damage macroscopically and histologically. Youssef et al. concluded that the protective effects of empagliflozin and metformin against dextran sulfate sodium-induced colitis occur through enhancing AMPK phosphorylation [46]. 

Other hypoglycemic meditations like glucagon-like peptide agonists (GLP-1) or dipeptidyl peptidase four inhibitors (DPP-4) have a role in the regulation of uncontrolled gut inflammation and immune response in diabetic patients and result in a lower incidence of hospital admissions and receiving steroid treatment in diabetic patients. GLPs can promote gut health through tissue repair of injured epithelium and better modulation of T cell differentiation, macrophages, and dendritic cells. GLPs can also reduce pro-inflammatory cytokines, which protect other extra-intestinal organs against the deleterious effects of inflammatory bowel disease. GLP-1-A medications can reduce the levels of different inflammatory biomarkers, such as CRP and TNF-α. GLP-1 and DPP-4 have shown significant evidence of ameliorating intestine inflammation in mice. In humans, GLP-1 and DPP-4 have been suggested to be used in IBDs to decrease gut inflammation in one cohort study [47], where 3751 patients with concomitant IBDs and DM 2 received anti-diabetic medications, 982 patients were on GLP-1 or DPP-4, and 2769 patients were receiving other medications. The adjusted incidence rate ratio of the composite outcome was 0.52. Hence, Villumsen et al. concluded that GLP-1-based therapies may improve the disease course of IBDs. A significant percentage of IBD patients do not respond to traditional therapy [47]. Hence, Zatorski et al. studied the role of a different class of GLP medications on IBDs and concluded that GLP-2 (teduglutide) might be much more promising than GLP-1, and also suggested use of both GLPs in simultaneously can have a synergistic effect in IBD treatment through stricter attenuation of gut immune and inflammatory response [48]. Ryan et al. compared the effect of metformin and DPP4 on gut microbiota in mice and concluded that either medication has excellent or harmful effects on different species of normal flora [49]. Hence, combining different oral anti-diabetic medications and certain probiotics can provide the best medium for normal flora to treat IBDs [50].

## Conclusions

Research on different models of diet and lifestyle and which model can help humankind regain the same physical and mental of ancient people is yet to come. The microbiome is a relatively new research field, and its correlation with different pathologies is being investigated extensively. The use of probiotics and postbiotics is getting more popular in treating gut diseases like IBDs and also as mood stabilizers. Intermittent fasting and healthy sleep hygiene are well advocated as simple but efficient gates to detoxify our bodies and help against IBDs. Other different natural interventions against IBDs have been studied separately, with few studies that combine few; for example, the addition of probiotics to metformin improved its side effects, and patients tolerated metformin better. Very few studies integrated a comprehensive approach that combines all the suggested natural methods in IBD patients. Since the etiology and the symptoms of IBDs are inflammatory-mediated, we highly suggest this comprehensive natural approach (FMT, probiotics, postbiotics, vitamins, healthy diet, intermittent fasting, high physical activity, good quality sleep, sun exposure, oral antidiabetics) that can move our bodies a step closer to our ancestors and possibly eliminate IBDs.

## Appendices

If you have Crohn's disease disease or ulcerative colitis, have you tried any of the above listed non-medication interventions? And did any of them work?
